# Defining the Sister Rat Mammary Tumor Cell Lines HH-16 cl.2/1 and HH-16.cl.4 as an *In Vitro* Cell Model for *Erbb2*

**DOI:** 10.1371/journal.pone.0029923

**Published:** 2012-01-10

**Authors:** Sandra Louzada, Filomena Adega, Raquel Chaves

**Affiliations:** 1 Center of Genomics and Biotechnology, Institute for Biotechnology and Bioengineering, University of Trás-os-Montes and Alto Douro (IBB/CGB-UTAD), Vila Real, Portugal; 2 Department of Genetics and Biotechnology, University of Trás-os-Montes and Alto Douro, Vila Real, Portugal; Instituto Nacional de Câncer, Brazil

## Abstract

Cancer cell lines have been shown to be reliable tools in genetic studies of breast cancer, and the characterization of these lines indicates that they are good models for studying the biological mechanisms underlying this disease. Here, we describe the molecular cytogenetic/genetic characterization of two sister rat mammary tumor cell lines, HH-16 cl.2/1 and HH-16.cl.4, for the first time. Molecular cytogenetic analysis using rat and mouse chromosome paint probes and BAC/PAC clones allowed the characterization of clonal chromosome rearrangements; moreover, this strategy assisted in revealing detected breakpoint regions and complex chromosome rearrangements. This comprehensive cytogenetic analysis revealed an increase in the number of copies of the *Mycn* and *Erbb2* genes in the investigated cell lines. To analyze its possible correlation with expression changes, relative RNA expression was assessed by real-time reverse transcription quantitative PCR and RNA FISH. *Erbb2* was found to be overexpressed in HH-16.cl.4, but not in the sister cell line HH-16 cl.2/1, even though these lines share the same initial genetic environment. Moreover, the relative expression of *Erbb2* decreased after global genome demethylation in the HH-16.cl.4 cell line. As these cell lines are commercially available and have been used in previous studies, the present detailed characterization improves their value as an *in vitro* cell model. We believe that the development of appropriate *in vitro* cell models for breast cancer is of crucial importance for revealing the genetic and cellular pathways underlying this neoplasy and for employing them as experimental tools to assist in the generation of new biotherapies.

## Introduction

Breast cancer is one the most commonly occurring cancers among women and has been described as a molecularly heterogeneous disease. Genetic studies of breast cancer rely on the use of primary tumors, paraffin-embedded samples or cell lines. Breast cancer cell lines present the great advantage of being readily available, and the full characterization of cell line models has been shown to provide valuable insights regarding the degree of complexity of the polygenetic etiology of breast cancer and the biological mechanisms that characterize this disease [1]. Chemically induced carcinogenesis of the rat mammary gland has been used extensively to investigate breast cancer. In rat models, the carcinogenic compound 7,12-dimethylbenz[a]anthrazene (DMBA) is frequently used to induce tumors, and DMBA-induced rat mammary tumors and sarcomas are useful cancer models [2], [3], [4]. Using the evolutionary conservation of gene segments as a guide, animal models, such as the rat, constitute powerful tools to decipher pathways and genes involved in tumorigenesis [4]. Moreover, researchers now have access to powerful web servers and databases in which syntenic regions can be easily identified and associated with a great amount of information regarding human and rat genetics. The available animal tumor cell lines are often poorly characterized from a cytogenetic/genetic point of view, reducing their usefulness as cell models.

Here, we present the molecular cytogenetic/gene expression characterization of two DMBA-induced rat mammary tumor cell lines: the HH-16 cl.2/1 fibrosarcoma cell line and the HH-16.cl.4 adenocarcinoma cell line. The choice of these cell lines was based on two factors: first, the reliability of both cell lines as models has been demonstrated in investigations of the effects of glucocorticoid hormones on cell morphology and proliferation and the stability of cultured rat cells after infection with Moloney murine sarcoma virus [5], [6], [7]; second, these cell lines are commercially available to the entire scientific community, and when they are properly characterized, they may constitute reliable cell models for breast cancer research.

Performing a chromosome count constitutes a mandatory step in the cytogenetic characterization of cell lines, allowing an overview of their genetic variability and stability. Of the two investigated cell lines, only HH-16 cl.2/1 presents low polyploidy levels, indicating a certain degree of stability, and for this reason, detailed cytogenetic characterization was restricted to this cell line. The methodology used in this study included fluorescent *in situ* hybridization with rat and mouse chromosome paint probes to identify chromosomal rearrangements, complemented with BAC/PAC clones that assisted in the accurate detection of the breakpoint regions of the rearrangements as well as complex chromosome abnormalities. The increase in the number of copies (determined with specific BAC clones) of the *Mycn* and *Erbb2* genes detected in this analysis was of particular note. The development and progression of cancer are characterized by a variety of genetic modifications in mechanisms that control genome stability, including alterations in oncogenes [8]. *ERBB2* oncogene amplification constitutes one of the most important genetic alterations associated with human breast cancer and was found to be correlated with poor patient prognosis by Slamon and colleagues [9]. *MYCN* oncogene amplification is characteristic of human neuroblastomas, being found in 20% of these childhood cancers, and has been observed to be involved in breast tumorigenesis, with up-regulation being detected in inflammatory breast cancer [10]. In the present study, the amplification status of the rat counterpart *Erbb2* and *Mycn* genes was analyzed in the HH-16 cl.2/1 and HH-16.cl.4 rat cell lines by fluorescent *in situ* hybridization, and the expression of these genes was assessed by real-time reverse transcription quantitative PCR (RT-qPCR) complemented and validated with an RNA fluorescent *in situ* hybridization (RNA FISH) analysis.

Abnormal patterns of DNA methylation have been found in several types of human cancer. DNA hypermethylation may result in gene expression silencing and loss of protein function as well as being associated with cancer progression [11]. Currently, epigenetic therapies aim to restore hypomethylation and to reverse gene silencing induced by hypermethylation [12]. A cytosine analogue established as a potent inhibitor of DNA methylation, 5-Aza-2′-Deoxicitidine (decitabine), [13] has been used in both preclinical models and in cancer patients [14]. However, global demethylation effects in tumor cells treated with this agent remain poorly understood. Early studies suggest that the loss of DNA methylation is a common event in tumorigenesis [15], [16]. To evaluate global genome demethylation effects on gene expression in the studied rat tumor cell lines, cells were treated with 5-Aza-2′-Deoxicitidine, and *Mycn* and *Erbb2* expression was subsequently determined.

The cytogenetic and genetic characterization of the HH-16 cl. 2/1 and HH-16.cl.4 rat mammary cell lines, complemented with expression profiling analysis of the *Mycn* and *Erbb2* oncogenes and verification of the influence of global demethylation on the expression of these genes validates the use of these cell lines as models for breast cancer research.

## Materials and Methods

### Cell culture and chromosome preparation

The HH-16 cl.2/1 and HH-16.cl.4 cell lines were obtained from the German Collection of Microorganisms and Cell Cultures (DSMZ). Both cell lines were established from ascitic fluid of the same female Sprague-Dawley rat with a mammary tumor produced by injection of cultured cells from a DMBA-induced mammary tumor. When injected into rats, HH-16.cl.2/1 cells have been found to produce fibrocarcinomas while HH-16.cl.4 cells generate adenocarcinomas. Both cell lines were grown in RPMI 1640 supplemented with 10% FCS, 1% 200 mM L-Glutamine and 1% of a Penincilin-Streptomycin antibiotic mixture (all from Gibco, Life Technologies). The HH-16.cl.4 cell medium was also supplemented with 1% 100 mM Sodium Pyruvate MEM (Gibco, Life Technologies). Both cultures were passaged at confluence using 0.25% trypsin (1×) with EDTA in Hanks' balanced salt solution (Gibco, Life Technologies). For both cell lines, metaphase chromosomes were obtained by treatment with colcemide (10 µg/ml, Invitrogen, Life Technologies) for 45 minutes followed by hypotonic solution (0,05 M KCl, 30 minutes, 37°C) and fixation with methanol∶acetic acid (3∶1), and the samples were then dropped onto microscope slides.

### GTD-banding

Air-dried slides from the HH-16 cl.2/1 cell line were aged at 65°C overnight and then subjected to standard G-banding procedures with trypsin [17]. DAPI was used for staining (instead of routine Giemsa staining) to obtain a better contrast [18]. Inversion of the DAPI color in Adobe Photoshop (version 7.0) revealed the chromosome G-banding pattern (GTD-banding, G-bands revealed by trypsin with DAPI).

### Chromosome painting

Chromosome paint probes from *Rattus norvegicus* (RNO) and *Mus musculus* (MMU) were kindly provided by Dr. Johannes Wienberg and Dra. Andrea Kofler from Chrombios GmbH, Germany. Chromosome-specific probes were labeled by DOP-PCR using the universal primers 6MW (for RNO paints) and F/S (for MMU paints) together with incorporation of digoxigenin-11-dUTP (Roche) or biotin-16-dUTP (Roche).

Fluorescent *in situ* hybridization experiments were performed according to [19]. RNO paint probes were hybridized to chromosomes from both the HH-16 cl.2/1 and HH-16.cl.4 cell lines while MMU paint probes were only hybridized to HH-16 cl.2/1 chromosomes. The most stringent post-hybridization wash was 50% formamide/2×SSC at 37°C, and probe detection was performed using antidigoxigenin-5′TAMRA (Roche) and FITC conjugated with avidin (Vector Laboratories).

### Probe construction from BAC/PAC clones and FISH

BAC and PAC clones were obtained from the BACPAC Resources Center from Children's Hospital Oakland Research Institute (http://bacpac.chori.org/). The acquired clones were RP31-262B4, CH230-208E5, RP31-202O5, RP31-039D3, CH230-10B5 (for rat chromosome 6); CH230-174M18, CH230-9A5, CH230-215E5, CH230-27O13, CH230-165C24, CH230-117H20 (for rat chromosome 15); and CH230-162I16, CH230-276G18 and CH230-305O21 (rat *Erbb2* predicted clones). DNA from the clones was purified using QUIAGEN Plasmid Purification Kit as recommended by the manufacture (QIAGEN) and labeled with tetramethyl-rhodamine-5-dUTP (Roche) by Nick Translation (Abbott) for 2 hours at 15°C. Labeled probes were precipitated with an excess of sonicated normal rat genomic DNA and dissolved in hybridization solution. FISH procedures were performed as described in the Chromosome Painting section using chromosome preparations of the HH-16 cl.2/1 and HH-16.cl.4 cell lines.

For rat *Erbb2*, three BAC clones were selected *in silico* using the NCBI Map Viewer online resource (http://www.ncbi.nlm.nih.gov/mapview/) and then tested for the presence of *Erbb2* and mapped by FISH (see Figure S1). Briefly, a rat *Erbb2* genomic sequence obtained from the Ensembl database (http://www.ensembl.org/) was used to design specific primers for the amplification of this gene in the three BAC clones. PCR was performed with purified plasmid DNA from rat BAC clones (as described above), and PCR products with the predicted sizes were excised from 1.2% agarose gels, purified and sequenced. FISH procedures were performed as described above.

### FISH image capture, processing and analysis

Chromosomes were observed using a Zeiss AxioImager Z1 microscope, and images were captured using an Axiocam MRm digital camera with LSM 510 software (version 4.0 SP2). Digitized photos were prepared in Adobe Photoshop (version 7.0); image optimization included contrast and color adjustments that affected the whole image equally. Karyotypes were constructed following the nomenclature for rat chromosomes described by Levan [20], and chromosome rearrangements were described according to ISCN (2009) [21].

### Gene amplification criteria

Gene amplification was calculated based on the ratio between the number of gene signals and the number of chromosomes harboring that gene. *Mycn* amplification was defined for *Mycn*/RNO6≥2 and *Erbb2* amplification by *Erbb2*/RNO10≥2, with 2 being the cut-off value for both. Rat PAC clone RP31-202O5 was used to identify the *Mycn* gene, rat BAC clone CH230-162I16 allowed detection of *Erbb2*, and rat paint probes were used to identify chromosomes 6 and 10. Additional copies of each gene, detected by FISH at levels equal to or no more than 4-fold higher (when compared with normal gene number) were considered to be a *Mycn* or *Erbb2* gain.

### RNA isolation and reverse transcription quantitative real-time PCR

Total RNA from rat cell lines was isolated using the mirVana Isolation Kit (Ambion) following the manufacturer's recommendations. Expression experiments were performed using the TaqMan® RNA-to-CT™ 1-Step Kit (Applied Biosystems). The TaqMan Gene Expression Assay Mixes (primer/probe sets) used were beta-actin (Rn00667869_m1) and glyceraldehyde-3-phosphate dehydrogenase (GAPDH, Rn01749022_g1) as reference genes and *Mycn* (Rn01473353) and *Erbb2* (Rn00566561_m1) as targets (all assays were from Applied Biosystems). The 20 µl reactions included 2 µl of RNA sample (50 ng/µl), 1 µl of the primer/probe assay mixture, 10 µl of PCR Master Mix, 0.5 µl of RT enzyme mix (Applied Biosystems) and 6.5 µl of DEPC-treated water. The reactions were carried out in a 96-well optical plate at 48°C for 15 min and 95°C for 10 min, followed by 40 cycles of 95°C for 15 s and 60°C for 1 min. PCR was carried out in the ABI 7500 Fast Real Time PCR system (Applied Biosystems). All reactions were performed in triplicate, and negative controls (without template) were run for each master mix. SDS software version 1.4 (Applied Biosystems) was applied for comparative analysis, and the relative expression level was normalized with multiple reference genes. The 2^−ΔΔCT^ method [22] was used to calculate fold changes in the expression levels of the genes of interest using a control RNO sample as a calibrator. Expression fold changes≥3 were considered relevant.

### Statistical analysis

Student's t-test was used to compare the data obtained. Values were expressed as the mean ± SD, and differences were considered statistically significant at p<0.05, representing the 95% confidence interval of the mean expression level.

### RNA fluorescent *in situ* hybridization

RNA FISH was performed using the QuantiGene ViewRNA plate-based assay kit (Panomics) following the manufacturer's recommendations with some modifications. Briefly, HH-16 cl.2/1 and HH-16.cl.4 cells were grown on polysine coated glass slides, fixed using 8% formaldehyde, dehydrated in ethanol (50%–70%–100%) and held at 4°C overnight. Then, cells were rehydrated, permeabilized and hybridized as recommended, except that protease digestion was optimized for each cell line. The RNA target was human *ERBB2* (Panomics), and the reference RNA was human/rat/mouse 18S RNA (Panomics). Confocal fluorescence images were captured on an LSM 510 META with a Zeiss Axio Imager Z1 microscope and LSM 510 software (version 4.0 SP2). For each scan, the same microscope settings were employed for all images to normalize the results. The lasers used were as follows: argon (488 nm) set at 12.9%, helium–neon (543 nm) set at 50.8% and Diode (405 nm) set at 9.9%. The pinhole was set to 96 µm (1.02 airy units) for argon laser, 102 µm (0.98 airy units) for helium–neon laser and 112 µm for the Diode laser using a 63× objective. Images were captured at a scan speed of 5 (3.30 µs) with 1 µm thick Z sections and processed using the “3D Viewer” plug-in for ImageJ. Twenty slide fields were randomly selected and analyzed by counting the number of signals in each cell.

### 5-Aza-2′-Deoxicitidine demethylation

For global genome demethylation, the media for the HH-16 cl.2/1 and HH-16.cl.4 rat cell lines were supplemented with different concentrations of 5-Aza-2′-Deoxicitidine (Sigma) (3 µM, 10 µM and 30 µM) for 72 hours. Every 24 hours, the medium was changed, followed by the addition of 5-Aza-2′-Deoxicitidine. After the 72 h period, a sample of the cells was collected for RNA extraction, and remaining cells were allowed to grow without drug treatment for another 72 hours, after which they were also subjected to RNA extraction. Additionally, the HH-16 cl.2/1 and HH-16.cl.4 cell lines were grown without 5-Aza-2′-Deoxicitidine as controls.

## Results

### HH-16 cl.2/1 and HH-16.cl.4 morphological features and ploidy

**Figure 1:**
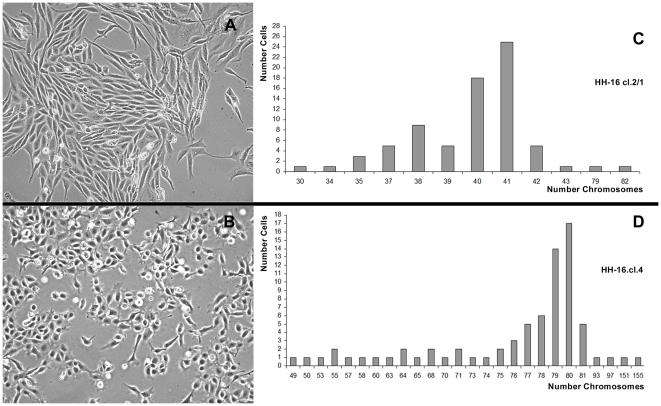
Morphology (×10) and ploidy of HH-16 cl.2/1 and HH-16.cl.4 cells. HH-16 cl.2/1 cell line presenting a fibroblastoid cell morphology with the cells growing in a criss-cross pattern (A), and HH-16.cl.4 cells morphology showing epitheloid shaped cells (B). Chromosome count analysis revealed a near-diploid karyotype with low level of polyploidy in HH-16 cl.2/1 (C) while a wide range of different chromosome numbers were observed in HH-16.cl.4, being the most representative the near-tetraploid karyotype (D).

Phase contrast microscopy analysis of the HH-16 cl.2/1 cell line revealed a fibroblastoid cell morphology, with the cells growing in a criss-cross pattern (Figure 1A). The HH-16.cl.4 line presented distinct cell morphology, with epitheloid-shaped cells growing in monolayer (Figure 1B).

Chromosome number analysis of the HH-16 cl.2/1 rat mammary fibrosarcoma cell line was carried out throughout the examination of 75 cells. The results show that this cell line presents a near diploid karyotype (Figure 1C), with 2n = 42 being the normal chromosome number for this species. The HH-16 cl.2/1 modal chromosome number is 40–41 (2n = 39–43 is the ploidy referenced in the available cell line description in the DSMZ database), and the polyploidy levels of this line are reduced (less than 3%), with only two cells being observed with a nearly tetraploid karyotype, containing 79 and 82 chromosomes. Chromosome number analysis was also performed for the HH-16.cl.4 rat mammary tumor cell line based on examination of 75 cells. This cell line presents a nearly tetraploid karyotype (Figure 1D) with a modal number of 79–80 (4n = 79–84 is the ploidy referenced in the available cell line description in the DSMZ database). When compared with the sister cell line, HH-16.cl.4 shows a wider range of cells with different chromosome numbers, with approximately 9% of cells being observed to have a nearly triploid karyotype (60–68 chromosomes) and 2% of cells exhibiting a nearly heptaploid karyotype (151–155 chromosomes). This variability in ploidy might be reflected in significant levels of karyotypic heterogeneity within this cell line, which are indicative of a higher order of complexity and instability when compared with the HH-16 cl.2/1 cell line. This observation restricted large-scale cytogenetic characterization to only the HH-16 cl.2/1 cell line, which apparently presents a more “stable” karyotype.

### Cytogenetic Characterization

#### Identification of clonal chromosome rearrangements

**Figure 2:**
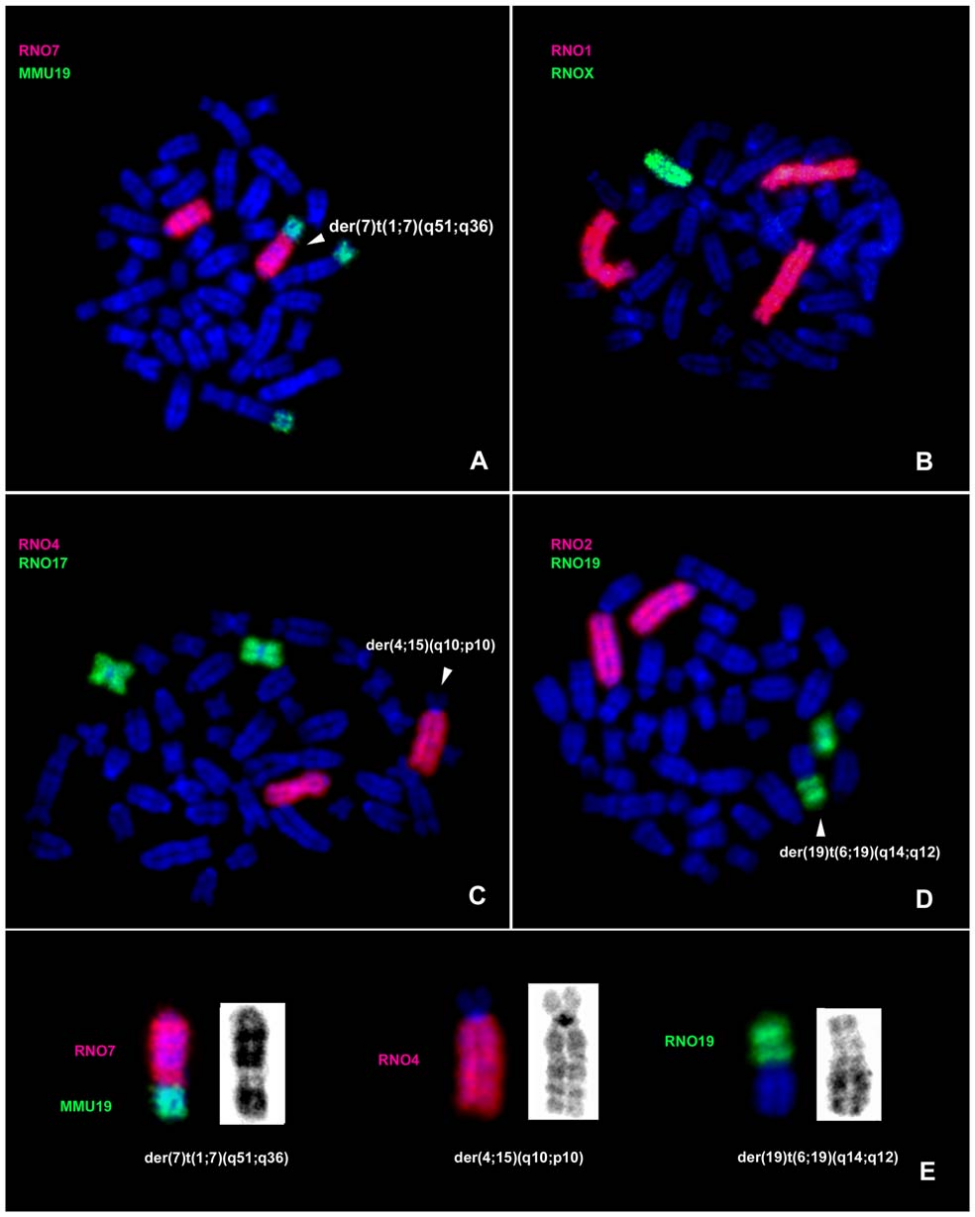
Molecular cytogenetic characterization of HH-16 cl.2/1 clonal chromosome rearrangements. Representative images of *in situ* hybridization with RNO and MMU paint probes onto HH-16 cl.2/1 metaphases (A–D), highlighting the derivative chromosomes. Derivative chromosomes are shown in detail (FISH and GTD) (E).

A combination of G-banding and fluorescent *in situ* hybridization was used in the cytogenetic characterization of clonal rearrangements for HH-16 cl.2/1. Paint probes for each rat chromosome (RNO1-20, X) and from mice (MMU19) were successfully hybridized to HH-16 cl.2/1 cell line chromosomes (Figure 2A–E), revealing a total of 13 rearrangements, both numeric and structural in character, involving chromosomes RNO1, RNO3, RNO4, RNO6, RNO7, RNO11, RNO13, RNO15, RNO18, RNO19 and RNOX. Three numerical changes were observed, involving a whole chromosome gain (+1) and two losses (−X, −18), with X chromosome monosomy being one of the most representative rearrangements. The rat chromosomes associated with greater numbers of rearrangements were RNO1, RNO6, RNO15 and RNO19. More structural than numerical aberrations were observed, and derivative chromosomes resulting from translocations were the predominant structural abnormalities. The most frequent structural chromosome rearrangements identified using this approach were as follows: t(3;11)(p12;p12), der(4;15)(q10;p10), der(7)t(1;7)(q51;q36), del(13)(p13) and der(19)t(6;19). Almost all rearrangements were unbalanced, involving gains and losses of chromosome segments. G-banding analysis allowed us to determine that the region of chromosome 1 involved in the rearrangement der(7)t(1;7)(q51;q36) was the terminal region. To confirm this analysis, the MMU19 paint probe was used because it is syntenic to this region in the rat. This approach confirmed that the region presented by the derivative chromosome is 1qter→1q51 (Figure 2E).

#### High-resolution chromosome rearrangement characterization and identification of breakpoint regions

**Figure 3:**
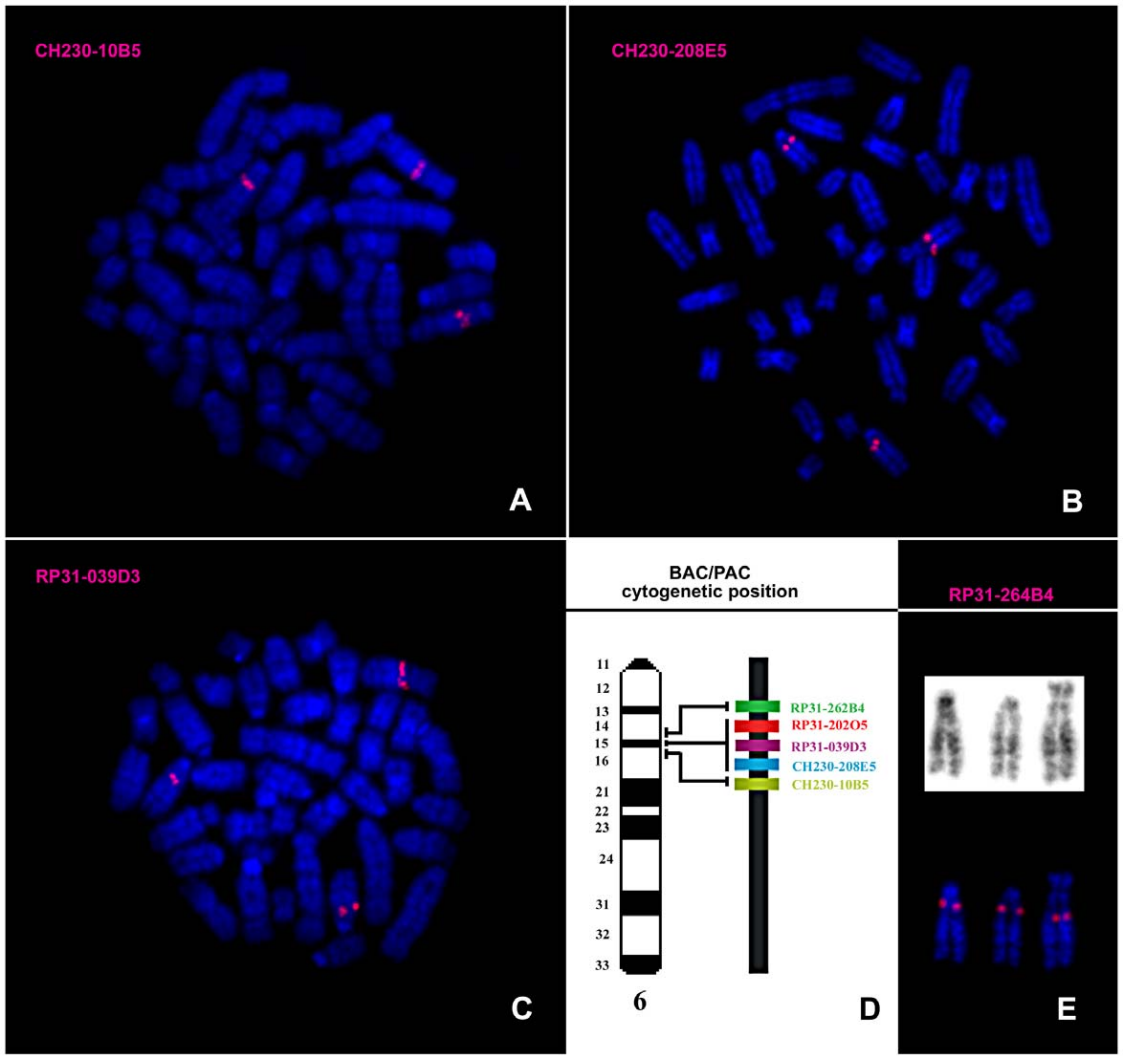
Molecular characterization of the rearrangements involving RNO6 using BAC/PAC clones. Representative images of *in situ* hybridization with BAC/PAC clones onto HH-16 cl.2/1 metaphases (A–C). Chromosome map of the region from bands 6q14 to 6q16, showing the relative positions of the clones used in this study (not to scale) (D). GTD and RP31-262B4 hybridization on the two normal RNO6 and one derivative chromosome of a rearranged cell (E).

**Figure 4:**
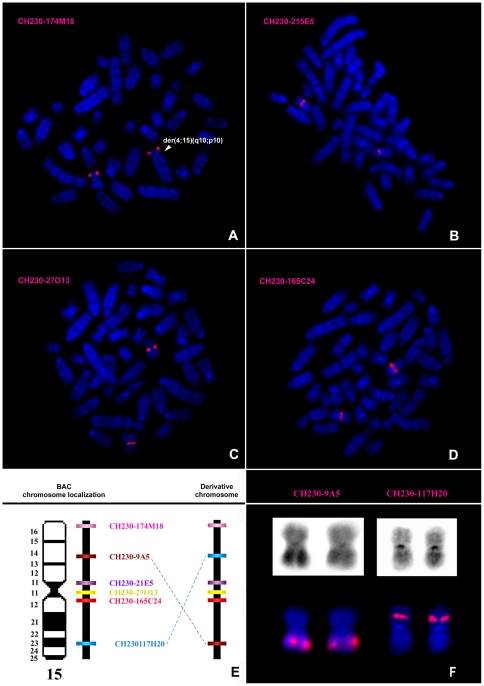
Molecular characterization of the rearrangements involving RNO15 using BAC clones. Representative images of *in situ* hybridization with the BAC clones onto HH-16 cl.2/1 metaphases (A–D). Chromosome map of RNO15 showing the relative positions of the clones used in this study, and the respective clone positions in the rearranged chromosome (not to scale) (E). GTD and CH230-9A5 and CH230-117H20 hybridization on the derivative chromosomes of a rearranged cell (F).

To refine the cytogenetic characterization, a total of 8 BAC and 3 PAC clones were hybridized to HH-16 cl.2/1 cell line chromosomes. The selected clones contained regions of rat chromosomes 6 (RP31-262B4, CH230-208E5, RP31- 202O5, RP31-039D3, CH230-10B5) and 15 (CH230-174M18, CH230-9A5, CH230-215E5, CH230-27O13, CH230-165C24, CH230-117H20), which were physically mapped in a previous study [23]. The BAC/PAC results allowed the identification of the breakpoint regions of the derivative chromosomes involving RNO6 to der(19)t(6;19)(q14;q12), der(19)t(4;19)(q31;p11)t(6;19)(q14;q12) and der(18;19)t(18;19)(p10,q10)t(6;19)(q14;q12), assigning the location of the breakpoint in all of these chromosomes to band 6q14, above the region included within clone RP31-262B4 (Figure 3). Concerning the analysis of RNO15, BAC mapping allowed the identification of these breakpoint chromosome regions involved in the der(4;15)(q10;p10) and der(15)del(15)(p11)t(1;15)(q12;q24) (Figure 4). Regarding the first derivative chromosome, it was possible to verify that it involved the entire chromosome 15p arm in a whole-arm translocation with chromosome 4 (Figure 4A). Concerning der(15)del(15)(p11)t(1;15)(q12;q24), BAC clones assisted in the identification of chromosome regions 15p11 and 15q24, which were involved in the formation of the derivative chromosome (Figure 4B–D). Moreover, the BAC analysis allowed the detection of a complex rearrangement in chromosome 15 that was not detectable using chromosome painting alone. After physical mapping of all of the BAC clones, two of them (CH230-117H20 and CH230-9A5) were found to have assumed different cytogenetic positions than expected (Figure 4E). The type of structural rearrangement that would most likely explain these results is a pericentric inversion. However, the remaining BAC clones used in this chromosome analysis were shown to assume the expected locations, meaning that the region between CH230-117H20 and CH230-9A5 maintained its expected order, suggesting a more complex rearrangement. We suggest the occurrence of a second pericentric inversion event involving two other breakpoints. Nevertheless, we cannot discard other possible events leading to the observed derivative chromosome. An interesting characteristic was that both RNO15 homologs, der(4;15)(q10;p10) and der(15)del(15)(p11)t(1;15)(q12;q24), present this configuration.

Integration of all of the FISH data allowed the construction of an HH-16 cl.2/1 composite karyotype based on the analysis of 64 cells:

30∼42,X,-X, +1 t(3;11)(p12;p12),der(4;15)(q10;p10),der(7)t(1;7)(q51;q36),del(13)(p13), der(15)del(15)(p11)t(1;15)(q12;q24,der(15)inv(15)(p14∼p16q23∼q25)inv(15)(p12∼p14q22∼q23)×2, −18,der(18)t(1;18)(q11;q12.3), der(18;19)t(18;19)(p10,q10)t(6;19)(q14;q12),der(19)t(6;19)(q14;q12), der(19)t(4;19)(q31;p11)t(6;19)(q14;q12)[cp64]

### Reconstruction of HH-16 cl.2/1 cell line clonal evolution

**Figure 5:**
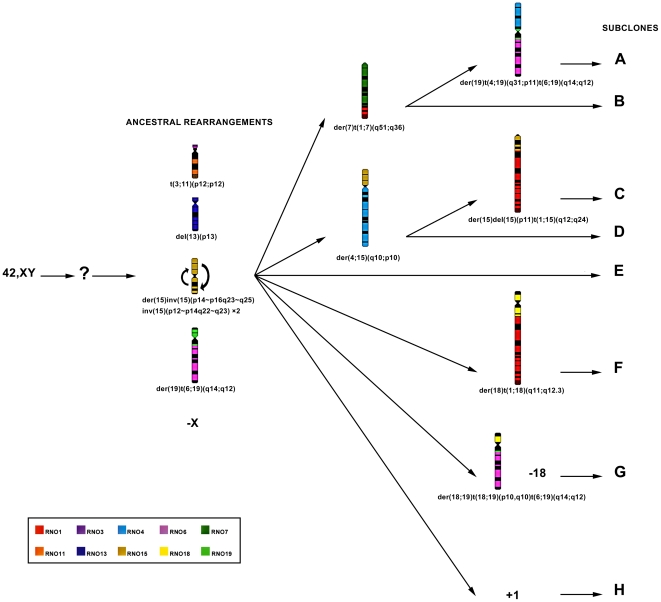
Chromosome reconstruction of the clonal evolution in HH-16 cl.2/1 tumor cell line. Diagram showing the hypothetic clonal evolution of HH-16 cl.2/1 chromosomes. In the diagram are shown numerical and structural clonal rearrangements. Ideograms represent all structural clonal rearrangements. Each rat chromosome is represented by a different color according to the legend. Subclones A–H are presently found in the cell line (respective karyotype formulas are shown in Table 1).

The chromosomal structural abnormalities t(3;11)(p12;p12), del(13)(p13), and der(15)inv(15)(p14∼p16q23∼q25)(p12∼p14q22∼q23) ×2 as well as the numeric change –X were observed in all of the cells analyzed (64 cells), suggesting a monoclonal origin of the tumor cell line. The other most frequent chromosomal abnormalities were der(19)t(6;19)(q14;q12), found in 45 cells; der(4;15)(q10;p10), found in 23 cells; and der(7)t(1;7)(q51;q36) observed in 20 of the 64 cells analyzed. This analysis permitted the identification of different cell subclones (Table 1), and comparison of these subclones allowed inferring ancestral rearrangements as well as a tentative reconstruction of the clonal evolution that occurred during tumor progression. The rearrangements present in all cells were considered to be part of the ancestral clone (as shown in Figure 5), from which several branches diverged during tumor progression (karyotype formulas presented in Table 1).

**Table 1:** Karyotypic formulas of the subclones (A to H) presently found in HH-16 cl.2/1 cell line.

Subclone	Karyotypic formulas
**A**	38∼42,X,-X,t(3;11)(p12;p12),der(7)t(1;7)(q51;q36),del(13)(p13),der(15)inv(15)(p14∼p16q23∼q25)inv(15)(p12∼p14q22∼q23)×2, der(19)t(4;19)(q31;p11)t(6;19)(q14;q12) [6]
**B**	35∼42,X,-X,t(3;11)(p12;p12),der(7)t(1;7)(q51;q36),del(13)(p13),der(15)inv(15)(p14∼p16q23∼q25)inv(15)(p12∼p14q22∼q23)×2, der(19)t(6;19)(q14;q12) [14]
**C**	30∼41, X,-X,t(3;11)(p12;p12),der(4;15)(q10;p10),del(13)(p13),der(15)del(15)(p11)t(1;15)(q12;q24), der(15)inv(15)(p14∼p16q23∼q25)inv(15)(p12∼p14q22∼q23)×2,der(19)t(6;19)(q14;q12) [9]
**D**	30∼41,X,-X,t(3;11)(p12;p12),der(4;15)(q10;p10),del(13)(p13),der(15)inv(15)(p14∼p16q23∼q25)inv(15)(p12∼p14q22∼q23)×2, der(19)t(6;19)(q14;q12) [14]
**E**	30∼42,X,-X,t(3;11)(p12;p12),del(13)(p13),der(15)inv(15)(p14∼p16q23∼q25)inv(15)(p12∼p14q22∼q23)×2, der(19)t(6;19)(q14;q12) [11]
**F**	39∼41,X,-X,t(3;11)(p12;p12),del(13)(p13), der(15)inv(15)(p14∼p16q23∼q25)inv(15)(p12∼p14q22∼q23)×2, der(18)t(1;18)(q11;q12.3),der(19)t(6;19)(q14;q12) [3]
**G**	38∼42,X,-X,t(3;11)(p12;p12),del(13)(p13),der(15)inv(15)(p14∼p16q23∼q25)inv(15)(p12∼p14q22∼q23)×2, der(18;19)t(18;19)(p10,q10)t(6;19)(q14;q12),−18 [4]
**H**	42,X,-X,t(3;11)(p12;p12),del(13)(p13),der(15)inv(15)(p14∼p16q23∼q25)inv(15)(p12∼p14q22∼q23)×2, der(19)t(6;19)(q14;q12),+1 [3]

The karyotype formulas correspond to the different subclones identified in HH-16 cl.2/1 cell line allowing the identification of ancestral chromosome rearrangements and to deduce the hypothetic clonal evolution shown in Figure 5.

### *In silico* analysis of breast cancer-related genes present in breakpoint regions

All of the identified breakpoint regions resulting from clonal chromosome rearrangements in the HH-16 cl.2/1 cell line are summarized in Figure S2. An *in silico* analysis using data from the Rat Genome Database (http://rgd.mcw.edu; assembly RGSC 3.4) and Ensembl (http://www.ensembl.org/; assembly RGSC 3.4) permitted screening of the breakpoint regions of the ancestral structural rearrangements for the presence of breast cancer-related genes (summarized in Table S1). With the exception of breakpoint bands 11p12, 15q22, 15q23, 15q24 and 15q25, all of the other breakpoints contain genes in the rat genome with human homologs that have been associated with breast cancer in humans.

### *Mycn* and *Erbb2* analysis

#### Gene amplification

**Figure 6:**
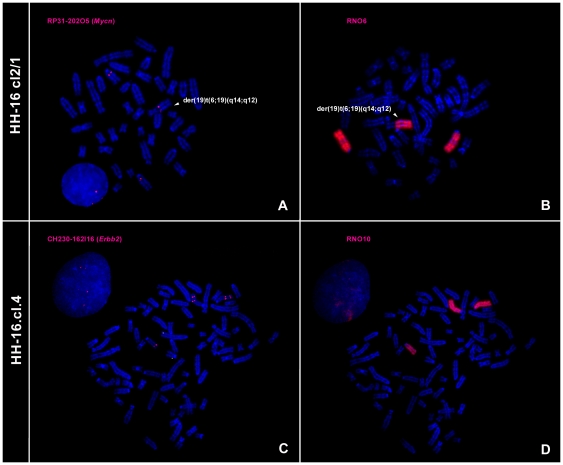
FISH results for *Mycn* and *Erbb2* amplification analysis in HH-16 cl.2/1 and HH-16.cl.4cell lines. Images show *Mycn* hybridizes in three chromosomes (A) two RNO6 and a derivative chromosome, der(19)t(6;19)(q14;q12) (B). Two *Erbb2* signals are present in chromosome 10 (C) identified with RNO10 paint probe hybridization (D).

Unlike most gene amplification studies using FISH, the present analysis was performed in metaphase chromosomes instead of interphase nuclei. This approach was advantageous, as it allowed a clear view of aneuploidies and chromosome rearrangements involving regions harboring the studied genes to be obtained. RNO6 painting and the rat PAC clone RP31- 202O5 were used to access the amplification status of *Mycn*. RP31-202O5 was earlier confirmed to contain *Mycn* gene and mapped to RNO 6q15.3-16 [23] and was also used in this work for the accurate identification of HH-16 cl.2/1 breakpoint regions. During HH-16 cl.2/1 cytogenetic characterization, it was possible to verify that this gene was present in three copies distributed among two intact RNO6 chromosomes and in the derivative chromosomes der(19)t(6;19)(q14;q12), der(18;19)t(18;19)(p10,q10)t(6;19)(q14;q12),der(19)t(6;19)(q14;q12) and der(19)t(4;19)(q31;p11)t(6;19)(q14;q12), indicating that a partial trisomy of RNO6 was involved in a translocation. The derivative chromosomes were not considered in the estimation of RNO6 for the *Mycn*/RNO6 calculation. As can be seen in Table 2, the most representative ratio was 1.5 (84.6%), corresponding to three *Mycn* signals distributed among two normal RNO6 chromosomes and one derivative chromosome (Figures 6A and 6B). *Mycn* was not considered to be amplified in this cell line, while a *Mycn* gain was considered to have occurred. The derivative chromosome der(19)t(6;19)(q14;q12) presenting *Mycn* was found in the majority of cells analyzed and, thus, was considered to represent an ancestral rearrangement (Figure 5). This finding raised the question of its importance in tumor initiation and progression, as this extra copy of *Mycn* was present in the ancestral clone. Regarding the HH-16.cl.4 cell line, all of the cells analyzed were characterized by a ratio of 1, presenting four *Mycn* signals distributed among four RNO6 chromosomes (data not shown). *Mycn* was also not amplified in the HH-16.cl.4 cell line.

**Table 2:** *Mycn* and *Erbb2* amplification and expression results for HH-16 cl.2/1 and HH-16.cl.4.

	*Mycn*		*Erbb2*		
Cell line	*Mycn*/RNO6	Expression Fold Change (±SD)	*Erbb2*/RNO10	Expression Fold Change (±SD)	RNA in situ signal Mean (±SD)
**HH-16 cl.2/1**	1.5 (84.6%)	−1.4 (±0.06)	1 (100%)	+2.6 (±0,1)	3.3 (±0.9)
	1.0 (15.4%)				
**HH-16.cl.4**	1.0 (100%)	+1.6 (±0.2)	1.7 (88.6%)	+10.7(±1,2)	15.2 (±3.6)
			1.5 (5.6%)		
			1.3 (2.9%)		
			1.0 (2.9%)		

*Mycn* and *Erbb2* amplification results were calculated as the *Mycn*/RNO6 and *Erbb2*/RNO 10 ratios, respectively (values between brackets represent the percentage of analyzed cells showing that result). Expression levels were accessed by RT-qPCR and RNA FISH (values between brackets represent the standard deviation).

To investigate *Erbb2* gene amplification, the RNO10 paint probe and CH230-162I16 rat BAC clone were used for FISH experiments with HH-16 cl.2/1 and HH-16.cl.4 chromosomes. This BAC clone was selected from a total of three clones acquired that were validated by PCR isolation followed by sequencing, with this clone being the only found to contain the *Erbb2* gene (Figure S1). CH230-162I16 was mapped by FISH for the first time in this study, and it was assigned to RNO 10q32.1, which is the cytogenetic position of rat *Erbb2* determined by Koelsch in 1998 [24]. According to the criteria used, no *Erbb2* amplification was detected in the HH-16 cl.2/1 cell line. The analysis revealed an *Erbb2*/RNO10 ratio of 1 in all analyzed cells (Table 2), corresponding to the presence of two *Erbb2* signals distributed among two RNO10 chromosomes (data not shown). Among the HH-16.cl.4 cells analyzed (Table 2), the most representative *Erbb2*/RNO10 ratio was 1.7 (88.6% of cells), which is near the cut-off value. In these cells, five *Erbb2* signals can be seen to be distributed among one intact RNO10 (one *Erbb2* signal) and two derivative RNO10 chromosomes with a duplication involving *Erbb2* loci (two *Erbb2* signals) (Figures 6C and 6D). As five *Erbb2* signals were observed, an *Erbb2* gain was considered to have occurred. An RNO10 polysomy was verified.

#### RNA expression analysis

**Figure 7:**
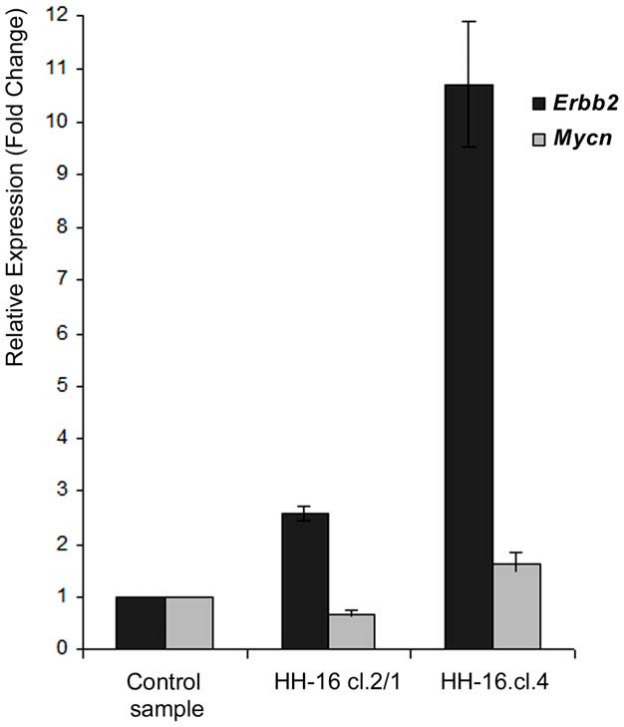
Relative expression of *Erbb2* and *Mycn* in the HH-16 cl.2/1 and HH-16.cl.4 cell lines. Expression results were obtained by reverse transcription quantitative real time PCR, normalized with the reference genes beta-actin and GAPDH and compared with a control sample. Data is presented as mean corresponding to fold change relative to the control sample (p<0.05). Error bars represent ±SD.

The levels of expression of the *Mycn* and *Erbb2* genes in HH-16 cl.2/1 and HH-16.cl.4 were determined by one-step real-time RT quantitative PCR (RT-qPCR), complemented and validated by RNA FISH (for *Erbb2*). Figure 7 shows the relative RT-qPCR quantification in terms of the fold change in *Erbb2* and *Mycn* RNA expression for both cell lines, which was normalized using multiple reference genes and is given relative to a calibrator (control RNO sample). All of the expression values presented in the graph were considered statistically significant following analysis using Student's *t-*test with a p value<0.05. Regarding *Mycn*, despite the statistical significance of the results, the fold changes in gene expression were low. For the HH-16.cl.4 cell line, a gain of 1.6 was verified, while for HH-16 cl.2/1, the expression value was below control sample expression (0.7 SD±0.06), corresponding to 1.4 times less expression than the control sample (Table 2). Only the results for *Erbb2* showed significant expression level changes, especially in HH-16.cl.4. The increase in *Erbb2* expression in HH-16 cl.2/1 was 2.6 fold (close to the cut-off value), and in HH-16.cl.4, *Erbb2* was expressed at a level 10.7 times higher than in the control sample (Table 2). HH-16 cl.2/1 *Erbb2* expression was approximately 4 times lower than in the sister cell line HH-16.cl.4, with significant expression only being found in the HH-16.cl.4 rat mammary cell line.

**Figure 8:**
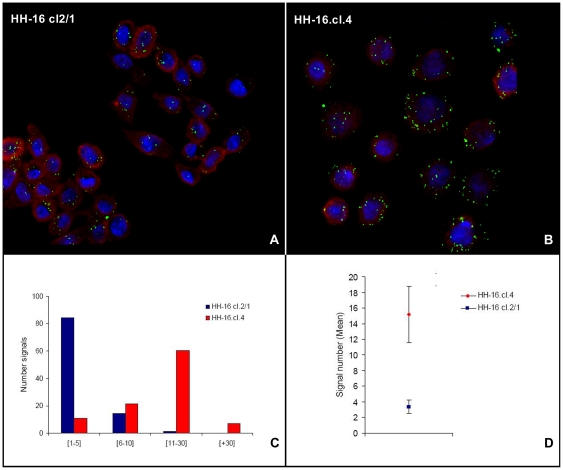
Expression analysis of *Erbb2* by RNA fluorescent *in situ* hybridization. RNA fluorescent *in situ* hybridization of *Erbb2* mRNA (green) and ribosomal 18S (red) used as reference, in HH-16 cl.2/1 (A) and HH-16.cl.4 (B) cell lines. The number of signals distributed by 4 intervals (C) and the mean number of signals for each cell line (error bars represents ±SD) (D) clearly showed differences in *Erbb2* expression between the two cell lines, being considerably higher in the HH-16.cl.4 cell line.

Evaluation of *Erbb2* expression was also performed using RNA fluorescent *in situ* hybridization in HH-16 cl.2/1 and HH-16.cl.4, validating the RT-qPCR analysis. This procedure allowed the visualization of *Erbb2* mRNA in individual cells of both cell lines (Figures 8A and 8B). The number of signals *per* cell was counted in 20 slide fields for each rat cell line, resulting in a total of 483 cells being analyzed for HH-16 cl.2/1 and 321 cells being analyzed for HH-16.cl.4. The results are displayed as the percentages of cells with total *Erbb2* signals falling between four numerical intervals: [1–5], [6–10], [11–30] and [+30]. Figure 8C shows that 84.5% of the HH-16 cl.2/1 cells present 1–5 *Erbb2* signals, and 60.4% of HH-16.cl.4 cells present 11–30 *Erbb2* signals, with these intervals being the most representative for each cell line. The mean number of signals *per* cell was 3.3 for HH-16 cl.2/1 and 15.2 for HH-16.cl.4 (Figure 8D and Table 2). These results show that there was higher expression of *Erbb2* in HH-16.cl.4 than in HH-16.cl.2/1, with 4.6 times higher expression being observed in the former cell line than in that latter, with is in accordance with the RT-qPCR data. The advantage of this methodology is the use of single cell analysis, which showed a wide range of expression in the cells of both cell lines. In addition to the expression analysis, RNA FISH permitted us to examine the sub-cellular localization of *Erbb2* mRNA. In both rat cell lines, *Erbb2* displayed cytoplasmic localization.

### Influence of 5-Aza-2′-Deoxicitidine global demethylation on *Mycn* and *Erbb2* RNA expression

**Figure 9:**
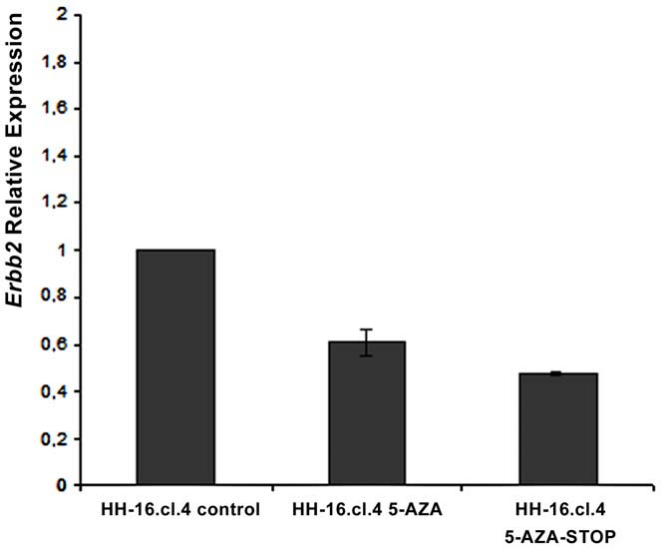
Relative expression analysis of *Erbb2* in HH-16.cl.4 cells after treatment with 5-Aza-2-Deoxicitidine. Relative expression analysis of *Erbb2* in HH-16.cl.4 cells treated with 5-Aza-2′-Deoxicitidine (HH-16.cl.4 5-AZA) and in HH-16.cl.4 cells after stopping the treatment with 5-Aza-2-Deoxicitidine (HH-16.cl.4 5-AZA-STOP). HH-16.cl.4 cells that were not treated with 5-Aza-2′-Deoxicitidine served as control (HH-16.cl.4 control). Data is presented as mean corresponding to fold change relative to control sample (p<0.05). Error bars represent ±SD.

Both cell lines were treated with 5-Aza-2′-Deoxicitidine for a period of 72 h, after which RNA was extracted and used to evaluate *Erbb2* and *Mycn* expression levels by means of RT-qPCR. These experiments were normalized with multiple reference genes (beta-actin and GAPDH) using RNA from cell lines that were not treated with 5-Aza-2′-Deoxicitidine as a control for calculating relative expression. No significant changes in *Mycn* expression were registered for either cell line. Statistically significant results based on Student's *t*-test (p value<0.05) were only obtained for *Erbb2* expression in HH-16.cl.4. *Erbb2* expression decreased after treatment with 5-Aza-2′-Deoxicitidine at a concentration of 3 µM in HH-16.cl.4 cells (Figure 9). This expression decrease, although significant (p<0.05), was not high when compared with the untreated cells. Moreover, *Erbb2* expression continued to decrease, even after the removal of the drug. These results show that global genomic demethylation only affects the expression of the *Erbb2* gene in the HH-16.cl.4 cell line, whereas it appears to have no effect on *Mycn* expression in either cell line.

## Discussion

A major opportunity to increase our knowledge regarding the biology of breast cancer is associated with the availability of experimental model systems that recapitulate the many forms of this disease. Recent studies have described the genetic characterization of breast cancer cell lines, showing their value in the investigation of the role of genomic alterations in cancer progression and as a resource for the discovery of new breast cancer genes [25], [26]. Rat cancer models, such as DMBA-induced rat tumors, have been found to be useful models for studying hormone-dependent breast cancer [1].

Here we present, for the first time, the genetic/cytogenetic characterization of two DMBA-induced rat mammary tumor cell lines, HH-16 cl.2/1 and HH-16.cl.4, which share the same genetic origin. These cell lines exhibit very distinct cytogenetic characteristics, beginning with different levels of ploidy. While HH-16.cl.4 cell line presents a nearly tetraploid karyotype, showing a wide range of cells with different chromosome numbers and levels of ploidy (Figure 1D), a nearly diploid karyotype with low levels of polyploidy can be found in HH-16 cl.2/1 (Figure 1C). This finding might be indicative of a higher order of complexity and chromosomal instability (CIN) of HH-16.cl.4, which is described as the presence of ploidy changes as well as high levels of aneuploidy [27]; these phenomena have been shown to have a direct causal role in tumorigenesis [28]. Additionally, heterogeneity reflects the existence of different tumor clones as well as a large number of apparently random chromosome changes, or so-called “cytogenetic noise”. For this reason, performing genome-wide cytogenetic characterization did not appear to be promising, and cytogenetic analysis was limited to the identification of relevant chromosome rearrangements associated with specific gene expression changes.

For the cytogenetic characterization of the HH-16 cl.2/1 cell line a multi approach was used which included G-banding, chromosome painting using rat and mouse probes and BAC/PAC clones hybridization. Clonal chromosome rearrangements were characterized (Figure 2) and specific breakpoint regions were identified (Figure 3 and 4). Few studies on the cytogenetic characterization of rat cell lines have been performed, particularly using rat or mouse paint probes. However, there have been some reports addressing rat tumor cell lines indicating RNO1 [3], [29], [30], RNO3 [29], RNO6 [31] and RNO15 [32], [33] as recurrent and/or relevant chromosomes related to the tumorigenesis/tumor progression in mammary fibrosarcomas, endometrial adenocarcinomas and lung cancer. Also in our study rearrangements in those chromosomes have been identified, as it is the case of the complex rearrangement involving RNO15 (Figure 4), only detected using the combination of varied cytogenetic tools. For this derivative chromosome we propose the occurrence of a double inversion as previously found in Acute Myeloid Leukemia karyotypes (e.g., [34], [35], [36]), and its presence in both RNO15 homologues can be explained by the loss of the normal chromosome, followed by the duplication of the abnormal homolog [37], [38], [39]. An interesting finding was the loss of an entire X chromosome which was present in all subclones identified. X chromosome loss has been described in numerous human cancer cases corresponding to the inactive X copy (e.g., [40], [41]) identified by a detectable Barr body (classic characteristic of X chromosome inactivation) [42], [43]. During our analysis of HH-16 cl.2/1 cells interphase nuclei, no Barr bodies were found in the X chromosome territory identified with the rat X paint probe (data not shown). This finding provides evidence that the X chromosome present in this cell line is the active X chromosome.

Assembly of the obtained data allowed us to deduce the clonal evolution of this tumor, which is illustrated in Figure 5. This diagram allows easy visualization of the ancestral and recent rearrangements, as well as providing an overview of the microevolutionary processes that have occurred in the progression of this tumor cell line. Analyses of karyotype clonal evolution have been performed previously in rats [32], [44], showing its relevance in the investigation of tumor progression. Moreover, the existence of ancestral structural chromosome abnormalities suggests a relevant role for these rearrangements in providing a selective advantage to this tumor cell line. An *in silico* analysis was performed focused on the breakpoint regions of the ancestral structural chromosome rearrangements and demonstrated that almost all of the breakpoint regions contain genes in the rat genome for which the human homolog has been associated with breast cancer (Table S1). This finding is relevant once translocations can lead to altered gene activity either through the formation of a chimeric gene product with cell transforming properties, or by juxtaposition of an oncogene with a foreign activator element [45].

In the cytogenetic characterization of HH-16 cl.2/1, the *Mycn* extra copy number was of particular note, especially because this characteristic was present in all of the cells analyzed and was considered to represent an ancestral condition. This observation raised the possibility of relevance of the *Mycn* gene in mammary tumor initiation and progression for both cell lines (once they are related). *MYCN* is part of a large family of oncogenes found to be amplified in human neuroblastomas and is correlated with aggressiveness and a negative prognosis in this type of pediatric cancer (reviewed by [46]). *Mycn* amplification has also been observed in rat tumors, specifically in uterine endometrial carcinomas [31], [47], however, the available literature does not include any investigation of *MYCN* amplification status in breast cancer. Overall, *Mycn* amplification was not detected in the HH-16 cl.2/1 or in HH-16.cl.4 cell lines, but an *Mycn* gain was found in HH-16 cl.2/1 (Figure 6 and Table 2). Additional copies of *MYCN* equal or less than 4-fold detected by FISH were considered as an *MYCN* gain, following a study on neuroblastoma [48].

The other gene analyzed in the present study was *Erbb2*. In humans, *ERBB2* gene amplification constitutes one of the most important genetic alterations associated with human breast cancer and was first correlated with poor patient prognosis by Slamon and colleagues [9]. Hence, no *Erbb2* amplification was found in the HH-16 cl.2/1 cell line, while for HH-16.cl.4 a low level of amplification was detected (Table 2). Chromosome painting data showed that *Erbb2* gain resulted from a chromosome alteration involving *Erbb2* gene locus resulting in its duplication (Figure 6C). Amplified DNA can be observed in various forms, including double minutes or amplified regions on a chromosome or distributed across the genome [49]. This gene gain may act as a precursor to further *Erbb2* amplification, or it may represent an alternative pathway for activating the oncogenic potential of this gene.

Generally, gene amplification has been associated with overexpression of the amplified gene(s) [49], although this correlation is not absolute. Both genes expression (*Mycn* and *Erbb2*) was accessed by RT-qPCR in the present work. *Mycn* RNA expression status showed no evidence for considerable expression changes which is in accord with the absence of gene amplification detected (Figure 7 and Table 2). These results also show that the *Mycn* gain corresponding to the three *loci* presented in the HH-16 cl.2/1 cell line was not reflected in an RNA expression change. With respect to *ERBB2*, the most frequently used method to determine its expression in breast cancer is immunohistochemistry (protein quantification) [50]. In human invasive duct carcinomas of the breast, erbB-2 protein overexpression is particularly frequent, and in most cases, this overexpression is caused by *ERBB2* gene amplification and associated with an unfavorable prognosis [9], [51]. Trastuzumab (Herceptin) is a humanized monoclonal antibody directed against the extracellular domain of the erbB-2 protein [52] that have been found to be effective when in presence of high levels of this protein [53], [54]. The *ERBB2* gene and erbB-2 protein status (gene amplification/protein overexpression) are considered useful markers for predicting the response to a specific cancer therapy, and analysis of these markers is mandatory for the identification of breast cancer patients that are amenable to trastuzumab treatment. In addition to immunohistochemistry, other methods have proven reliable in determining *ERBB2* expression status, such as real-time reverse transcription quantitative PCR [55], [56]. In the present study, a 3-fold increase in expression was considered to represent a significant expression change [57]. Relevant RNA expression changes for *Erbb2* were detected only for HH-16.cl.4 (10.7-fold increase) (Figure 7 and Table 2). This result correlates with the *Erbb2* gene gain, suggesting that the amplification, while low, may have played a role in the overexpression of *Erbb2* RNA in this cell line, although it may not be the only mechanism involved. The involvement of human chromosome 17 (harbors *ERBB2*) polysomy in erbB-2 protein expression has been discussed with some controversy [58]; however, some authors point to it as the cause of *ERBB2* overexpression [59], [60]. This cell line presents different levels of ploidy, and most of the cells analyzed present three copies of RNO10 (Figure 6D), suggesting the possible correlation of this chromosome copy number with the observed *Erbb2* expression levels. Another possible explanation is transcriptional regulation, which could have promoted the accumulation of *Erbb2* mRNA in the absence of high levels of amplification. Moreover, both older and more recent studies show that *ERBB2* RNA overexpression does not always correspond to erbB-2 protein overexpression, suggesting the existence of post-transcriptional regulation of *ERBB2* [61], [62], which shows the relevance of using RT-qPCR in routine assessment of *ERBB2* overexpression in human breast cancer in the clinical laboratory setting.

RNA FISH was used to measure *Erbb2* expression, complementing and validating the results of the RT-qPCR analysis. RNA fluorescent *in situ* hybridization is advantageous because it allows analysis of spatial gene expression patterns at a single-cell resolution [63], [64], [65]. This approach allowed clear visualization and semi-quantification of mRNA molecules in the cytoplasm, allowing quantification of the expression of *Erbb2* in both cell lines. The RNA FISH data strongly supported the RT-qPCR expression results, showing higher expression of *Erbb2* in HH-16.cl.4 (4.6 times greater) compared with the sister cell line HH-16 cl.2/1 (Figure 8 and Table 2), demonstrating to be an excellent technology when applied either alone or together with other technique.

Interestingly, the expression of *Erbb2* in the HH-16.cl.4 rat cell line appears to be affected by global genome demethylation. In the present study, HH-16 cl.2/1 and HH-16.cl.4 cells were treated with 5-Aza-2′-Deoxicitidine, promoting global genome demethylation. Statistically significant results were obtained for the *Erbb2* gene in the HH-16.cl.4 cell line, although the variation was not especially large (Figure 9). It has been demonstrated that *ERBB2* gene is overexpressed and unmethylated (in its promoter) in tumors and tumor cell lines, such as ovarian tumoral tissues and MCF-7 cell line [66], [67]. A similar study to ours was performed in a rat chondrosarcoma cell line, in which an increase in *Erbb2* expression was found after global genome demethylation [68]. Intriguingly, our data shows a decrease in *Erbb2* expression after 5-Aza-2′-Deoxicitidine treatment. While in the rat chondrosarcoma cell line, *Erbb2* promoter unmethylation seems to be the main cause for *Erbb2* overexpression, our data suggests a different pivotal epigenetic mechanism underlying the expression of this gene. Candidate negative regulators of *Erbb2* might be non-coding RNAs that for instance promote the degradation of transcripts [69]; or even other less understood epigenetic mechanisms such as splicing regulation [70] can explain our results. Our findings emphasize that future studies are mandatory to reveal the exact epigenetic events involved in the regulation of *Erbb2* expression, and that HH-16.cl.4cell line is an excellent tool to complete this task.

The cell lines used in the present work were generated simultaneously from the DMBA-induced rat mammary tumor [5], but despite having the same initial genetic background, fibroblastoid H-16 cl.2/1 cell line apparently reflect mesenchymal cells of the stromal part of the tumor, while the epitheloid HH-16.cl.4 cell line display epithelial origin. The cell lines different lineage, associated with the higher chromosomal instability revealed by HH-16.cl.4 (explaining the *Erbb2* overexpression here observed), suggests different mechanisms involved in tumor progression of both cell lines. In fact, HH-16.cl.4 exhibits a mainly tetraploid number of chromosomes. Tetraploidy can arise through a number of mechanisms, including cell fusion, mitotic slippage and cytokinesis failure [71]. In addition, tetraploid cells typically contain twice the normal complement of centrosomes that promote aberrant mitotic divisions and chromosome missegregation at a high frequency. Moreover, tetraploidy has been shown to initiate chromosomal instability and has been found to precede the development of CIN and aneuploidy in several cancers (e.g. [72], [73]). On the other hand, in the fibroblastoid H-16 cl.2/1 cell line, chromosome structure instability (CSI) seems to be the distinguishing feature, whose mechanisms are now starting to be disclosed [74]. Nevertheless, it seems that CSI can be the result of errors in the DNA damage checkpoints, DNA repair pathways, and/or mitotic segregation errors. However, mutations in proteins that permit cell cycle progression in the presence of double stranded breaks (e.g. p53, BRCA1, BRCA2, ATM and ATR) may also facilitate CSI [75].

In conclusion, molecular cytogenetics, gene expression profiling and examination of the influence of global demethylation on gene expression were used to characterize two rat mammary cell lines, H-16 cl.2/1 and HH-16.cl.4. All the presented results provide a platform for future studies on tumor progression and encourage the use of these cell lines as a model. In particular this study highlights H-16 cl.2/1 and HH-16.cl.4 potential as models for studying *Erbb2* associated mechanisms and as experimental tools to assist in the generation of new biotherapies.

We believe that the development of capable *in vitro* models of human breast cancer is of crucial importance in the study of cancer and, consequently, in the development of new therapeutics. We are confident that his work has contributed to the validation of this cellular model and to its use in future studies.

## Supporting Information

Figure S1 **Representative images of the** ***in situ*** **hybridization of putative** ***Erbb2*** **BAC clones onto RNO metaphases.** Both CH230-276G18 (A) and CH230-305O21 (B) hybridize in different locations than the *Erbb2* position determined by [24]. Only CH230-162I16 hybridizes at the cytogenetic position of *Erbb2* in *Rattus norvegicus* (10q32.1) (C). PCR amplification of *Erbb2* in the three clones (D). Only for CH230-162I16 BAC clone the expected 350 bp band is observed.
(PDF)

Figure S2 **Chromosomal location of the clonal rearrangements breakpoint regions in HH-16 cl.2/1 cell line.** Clonal rearrangements breakpoint regions in HH-16 cl.2/1cell line are displayed in the rat ideogram [20]. Each type of rearrangement originated by the breakpoints is identified by a specific color.
(PDF)

Table S1 ***In silico*** **analysis of breast cancer related genes present in the most representative rat breakpoint regions, and its correspondent human homolog.**
(PDF)
