# Supplementary material for: Defining the Sister Rat Mammary Tumor Cell Lines HH-16 cl.2/1 and HH-16.cl.4 as an In Vitro Cell Model for Erbb2
Source: PLoS One. 2012 Jan 10;7(1):e29923. doi: 10.1371/journal.pone.0029923 (PMC3254647; doi:10.1371/journal.pone.0029923)
Supplement: Figure S1 — Representative images of the in situ hybridization of putative Erbb2 BAC clones onto RNO metaphases. Both CH230-276G18 (A) and CH230-305O21 (B) hybridize in different locations than the Erbb2 position determined by [24]. Only CH230-162I16 hybridizes at the cytogenetic position of Erbb2 in Rattus norvegicus (10q32.1) (C). PCR amplification of Erbb2 in the three clones (D). Only for CH230-162I16 BAC clone the expected 350 bp band is observed. (PDF) [file pone.0029923.s001.pdf]

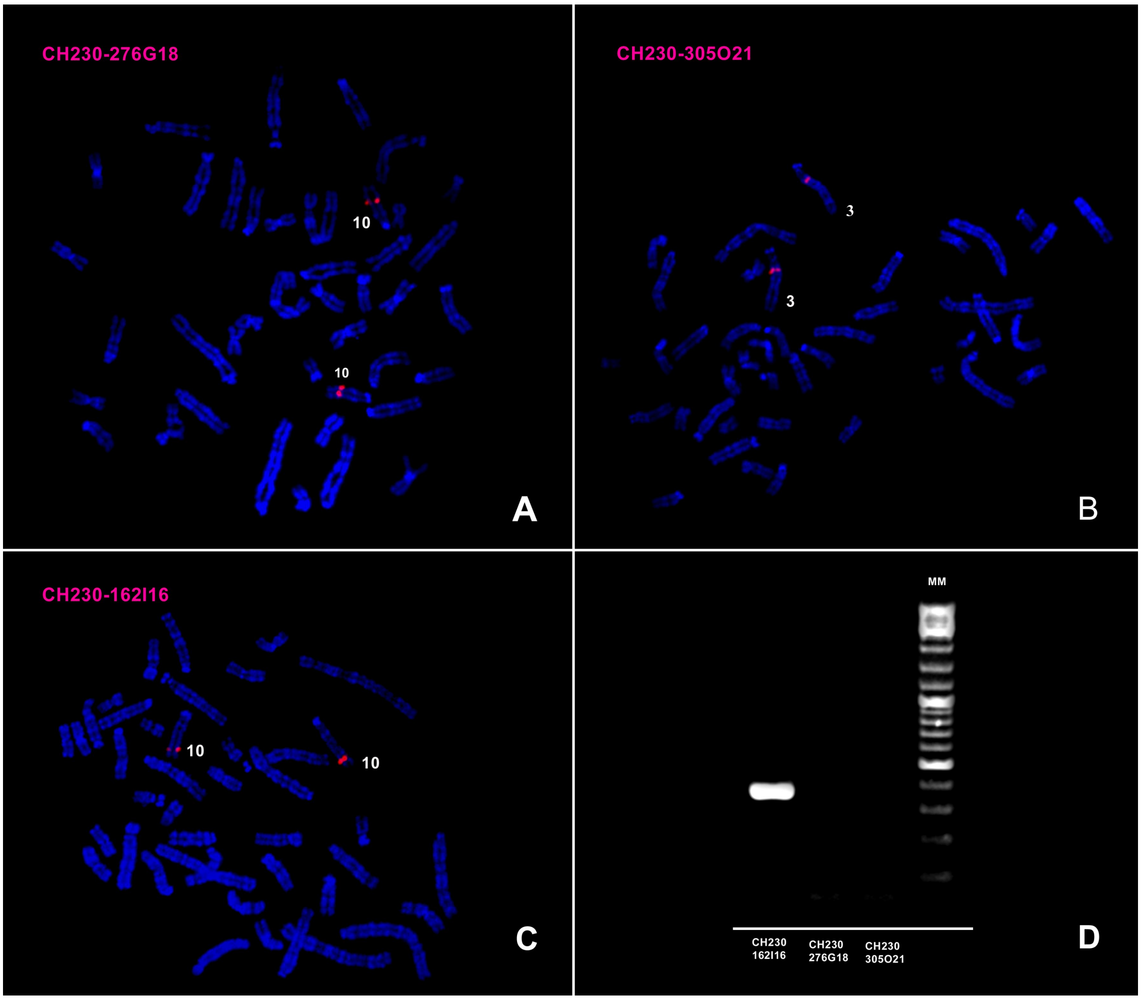

Figure S1. Representative images of the *in situ* hybridization of putative *Erbb2* BAC clones onto RNO metaphases. Both CH230-276G18 (A) and CH230-305O21 (B) hybridize in different locations than the *Erbb2* position determined by [24]. Only CH230-162I16 hybridizes at the cytogenetic position of *Erbb2* in *Rattus norvegicus* (10q32.1) (C). PCR amplification of *Erbb2* in the three clones (D). Only for CH230-162I16 BAC clone the expected 350 bp band is observed.
