# Supplementary material for: Defining the Sister Rat Mammary Tumor Cell Lines HH-16 cl.2/1 and HH-16.cl.4 as an In Vitro Cell Model for Erbb2
Source: PLoS One. 2012 Jan 10;7(1):e29923. doi: 10.1371/journal.pone.0029923 (PMC3254647; doi:10.1371/journal.pone.0029923)
Supplement: Figure S2 — Chromosomal location of the clonal rearrangements breakpoint regions in HH-16 cl.2/1 cell line. Clonal rearrangements breakpoint regions in HH-16 cl.2/1cell line are displayed in the rat ideogram [20]. Each type of rearrangement originated by the breakpoints is identified by a specific color. (PDF) [file pone.0029923.s002.pdf]

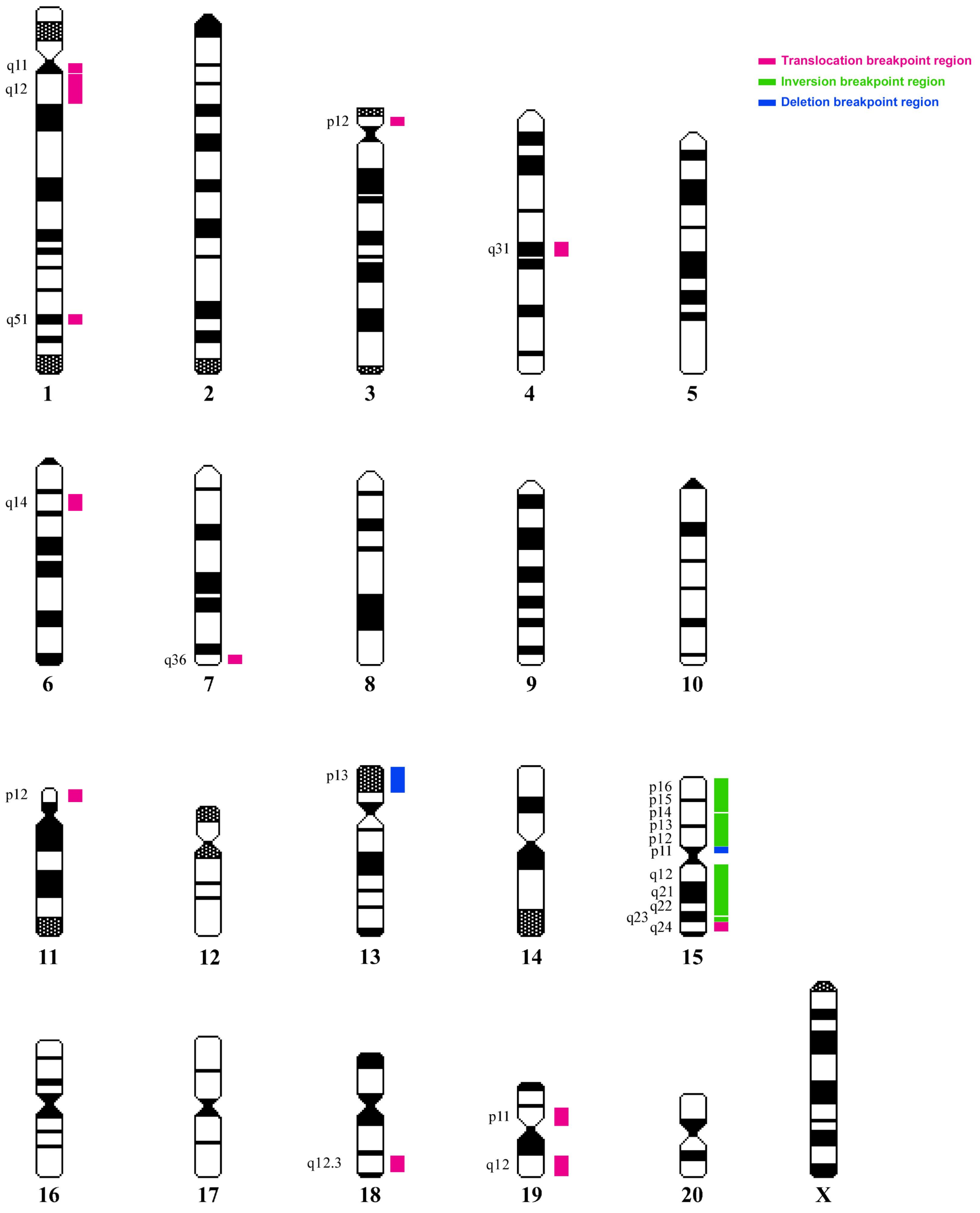

Figure S2- Chromosomal location of the clonal rearrangements breakpoint regions in HH-16 cl.2/1 cell line. Clonal rearrangements breakpoint regions in HH-16 cl.2/1cell line are displayed in the rat ideogram [20]. Each type of rearrangement originated by the breakpoints is identified by a specific color.
