# Supplementary material for: Defining the Sister Rat Mammary Tumor Cell Lines HH-16 cl.2/1 and HH-16.cl.4 as an In Vitro Cell Model for Erbb2
Source: PLoS One. 2012 Jan 10;7(1):e29923. doi: 10.1371/journal.pone.0029923 (PMC3254647; doi:10.1371/journal.pone.0029923)
Supplement: Table S1 — In silico analysis of breast cancer related genes present in the most representative rat breakpoint regions, and its correspondent human homolog. (PDF) [file pone.0029923.s003.pdf]

Table S1: *In silico* analysis of breast cancer related genes present in the most representative rat breakpoint bands, and its correspondent human homolog.

RNO		Human		
**Breakpoint region**	**Gene**	**Homolog gene**	**Cytogenetic position**	**Gene Definition**
3p12	*Rxra*	*RXRA*	9q34.2	retinoid X receptor, alpha
	Ptges	*PTGES*	9q34.11	prostaglandin E synthase
6q14	*Rhob*	*RHOB*	2p24.1	ras homolog gene family, member B
	Ncoa1	*NCOA*	2p23.3	nuclear receptor coactivator 1
	Cad	*CAD*	2p23.3	carbamoyl-phosphate synthetase 2, aspartate transcarbamylase, and dihydroorotase
13p13	*Bcl2*	*BCL2*	18q21.33	B-cell CLL/lymphoma 2
15p12	*Ctsb*	*CTSB*	8p23.1	cathepsin B
	Gnrh1	*GNRH1*	8p21.2	gonadotropin-releasing hormone 1 (luteinizing-releasing hormone)
	Ptk2b	*PTK2B*	8p21.2	PTK2B protein tyrosine kinase 2 beta
15p13	*Mmp14*	*MMP14*	14q11.2	matrix metallopeptidase 14 (membrane-inserted)
15p14	*Bmp4*	*BMP4*	14q22.2	bone morphogenetic protein 4
	Apex	*APEX*	14q11.2	APEX nuclease (multifunctional DNA repair enzyme) 1
	Ang1	*ANG1*	14q11.2	angiogenin, ribonuclease, RNase A family, 5
15p16	*Anxa7*	*ANXA7*	10q22.2	annexin A7
	Thrb	*THRB*	3p24.2	thyroid hormone receptor, beta
19q12	*Cdh1*	*CDH1*	16q22.1	Cadherin 1, type 1, E-cadherin (epithelial)
	Zfhx3	*ZFHX3*	16q22.2	Zinc finger homeobox 3
	Bcar1	*BCAR1*	16q23.1	Breast cancer anti-estrogen resistance 1
	Cdh13	*CDH13*	16q23.3	Cadherin 13, H-cadherin (heart)
